# Chondroitin Sulfate/Hyaluronic Acid-Blended Hydrogels Suppress Chondrocyte Inflammation under Pro-Inflammatory Conditions

**DOI:** 10.1021/acsbiomaterials.4c00200

**Published:** 2024-04-18

**Authors:** Michael Nguyen, Carly M. Battistoni, Paulina M. Babiak, Julie C. Liu, Alyssa Panitch

**Affiliations:** † Department of Biomedical Engineering, University of California, Davis, California 95616, United States; ‡ Davidson School of Chemical Engineering, Purdue University, West Lafayette, Indiana 47907, United States; § Weldon School of Biomedical Engineering, Purdue University, West Lafayette, Indiana 47907, United States; ∥ Wallace H. Coulter Department of Biomedical Engineering, Georgia Institute of Technology and Emory University, Atlanta, Georgia 30332, United States

**Keywords:** glycosaminoglycan, collagen, osteoarthritis, cartilage, tissue engineering

## Abstract

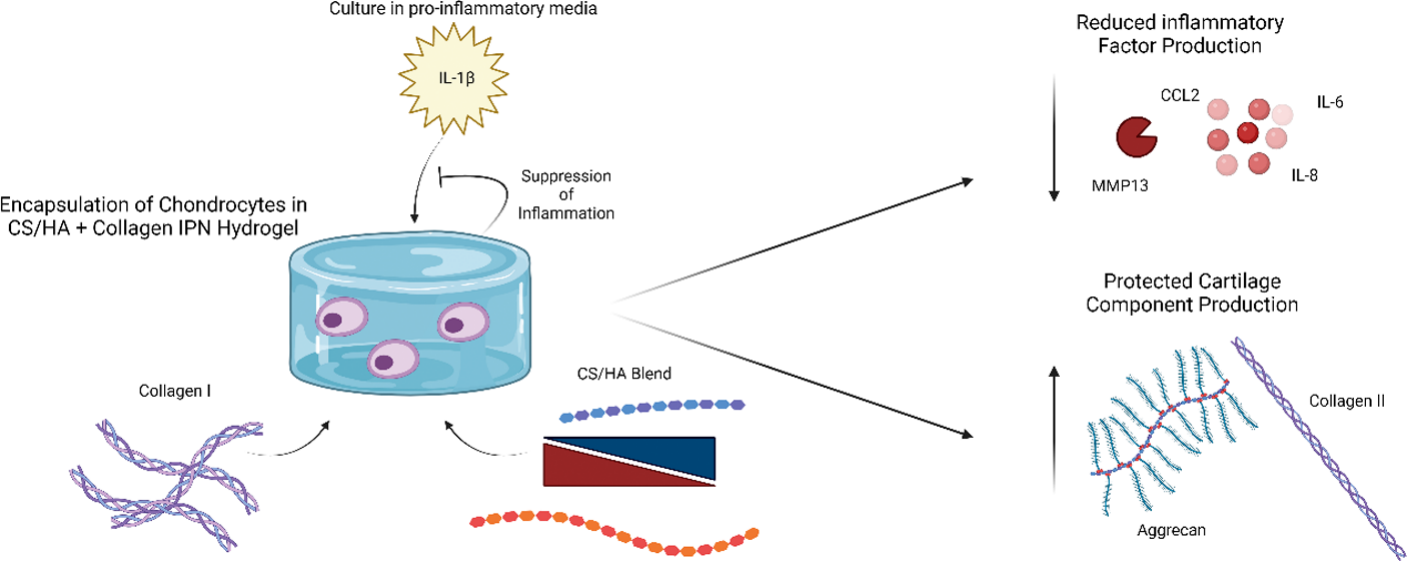

Osteoarthritis is characterized by enzymatic breakdown of the articular cartilage via the disruption of chondrocyte homeostasis, ultimately resulting in the destruction of the articular surface. Decades of research have highlighted the importance of inflammation in osteoarthritis progression, with inflammatory cytokines shifting resident chondrocytes into a pro-catabolic state. Inflammation can result in poor outcomes for cells implanted for cartilage regeneration. Therefore, a method to promote the growth of new cartilage and protect the implanted cells from the pro-inflammatory cytokines found in the joint space is required. In this study, we fabricate two gel types: polymer network hydrogels composed of chondroitin sulfate and hyaluronic acid, glycosaminoglycans (GAGs) known for their anti-inflammatory and prochondrogenic activity, and interpenetrating networks of GAGs and collagen I. Compared to a collagen-only hydrogel, which does not provide an anti-inflammatory stimulus, chondrocytes in GAG hydrogels result in reduced production of pro-inflammatory cytokines and enzymes as well as preservation of collagen II and aggrecan expression. Overall, GAG-based hydrogels have the potential to promote cartilage regeneration under pro-inflammatory conditions. Further, the data have implications for the use of GAGs to generally support tissue engineering in pro-inflammatory environments.

## 1 Introduction

Osteoarthritis (OA) is one of the most common and significant joint diseases in the world, with factors such as age, obesity, and genetics contributing to its prevalence.^1,2^ OA is a degenerative disease of the joint, characterized by the disruption in the homeostasis of the articular cartilage, breakdown of the extracellular matrix, and dysfunction of the resident chondrocytes.^3^ While it was previously thought that much of the degradation of cartilage in OA was due to mechanical wear, research in recent decades has highlighted the importance of pro-inflammatory cues such as interleukin 1β (IL-1β)^4^ and tumor necrosis factor alpha (TNF-α)^5^ in the context of total joint inflammation.^6−9^ Pro-inflammatory cues have been found in the synovial fluid of OA-affected joints, albeit at lower concentrations than those found in rheumatoid arthritic joints, and these cytokines are now believed to play a significant role in the progression of OA.^10,11^

In late-stage OA, excessive degradation of the articular cartilage can lead to the formation of significant-sized cartilage defects, leading to pain and loss of limb movement.^12^ Due to the avascular nature of cartilage, as well as the low resident cell population, cartilage has a limited healing capability.^13,14^ Treatments like autologous chondrocyte implantation (ACI), where the cartilage defect is repopulated with the patient’s own chondrocytes to repair the defect, have seen some clinical success, though the quality of the new cartilage is still inferior to that of the original.^15^ Furthermore, adverse effects such as graft failure, fibrosis, and hypertrophy have been observed following the ACI procedure.^16^ These adverse effects have been correlated with increased synovial fluid levels of IL-1β, suggesting that inflammatory activity may be the cause of the procedure failure.^16−18^

To address the issue of failed cartilage defect healing, the field of tissue engineering has sought to develop methods to engineer cartilage to repair defects.^19−22^ Although a diverse set of materials has been developed and produced robust cartilage replacements in vitro, many of these studies were performed in the absence of pro-inflammatory cytokines. As such, few studies have examined the potential effects of pro-inflammatory cytokines on the outcomes of engineered cartilage. This is an important consideration, as exposure to pro-inflammatory cytokines that exist in the joint environment can significantly affect the chondrocytes’ ability to produce new matrix components and can shift them into a catabolic state. Specifically, there can be increased secretion of collagenases like matrix metalloproteinase 13 (MMP13),^23^ aggrecanases like a disintegrin and metalloproteinase with thrombospondin motifs 4 and 5 (ADAMTS4, ADAMTS5),^24^ and hyaluronidases. Conversely, many studies have examined dampening the effects of inflammation on chondrocytes through methods such as the addition of anti-inflammatory soluble factors and cell cocultures, but many of these studies focused on the inflammatory response of chondrocytes alone, rather than their anabolic capability.^25−30^ Although both aspects of OA treatment, the repair of defects and the dampening of inflammation, have been well studied, there are few studies that have examined the overlap of tissue engineering and the pro-inflammatory environment.

To fill this knowledge gap, we sought to study the engineering of cartilage replacement using chondrocytes encapsulated in a hydrogel scaffold to support anabolic activity while also protecting the cells from a pro-inflammatory environment. To accomplish this, we employed glycosaminoglycans (GAGs), chondroitin sulfate (CS), and hyaluronic acid (HA) as polymers. The use of these GAGs for a cell scaffold provides two main benefits: promotion of chondrogenic activity^25,31−34^ and anti-inflammatory effects.^35−39^ Many studies have examined one aspect, but few studies have examined both aspects in one study. For example, previous research has demonstrated that the incorporation of these GAGs, either as scaffold components^32,40^ or in their soluble form,^41^ leads to better outcomes with regard to cartilage tissue engineering. Also, many studies have demonstrated the anti-inflammatory effects of HA and CS in their soluble form against different pro-inflammatory agents.^26,27,38^ In this study, we went beyond looking at a single attribute. To do so, we modified HA and CS minimally to prevent the loss of biological activity. Because our previous work demonstrated the superiority of a blended GAG hydrogel over a homopolymer hydrogel of either CS or HA,^42^ we used hydrogels comprising a blend of CS and HA in this study. Two GAG ratios were used to probe for any differences in outcomes based on relative GAG levels. Finally, some hydrogel groups contained collagen to form GAG/collagen type I interpenetrating network (IPN) hydrogels. In these IPN gels, collagen adds a tensile force to oppose the swelling force of the GAG hydrogel, reduces fluid infiltration and volumetric expansion, and adds binding sites for the cells.^43^ Altogether, the study of GAG hydrogels with different GAG blends and collagen contents demonstrated an interplay between the cells and the bioactive scaffold that has implications in the context of tissue engineering in a pro-inflammatory environment.

## 2 Materials and Methods

### 2.1 Synthesis and Characterization of Thiolated Hyaluronic Acid and Chondroitin Sulfate

Thiolated GAGs were synthesized using a previously reported method.^42^ HA (molecular weight 100 kDa, Lifecore Biomedical) and CS (molecular weight 40 kDa, Seikigaku Corporation) were first dissolved in 0.1 M 2-(*N*-morpholino)ethanesulfonic acid (MES) buffer with 0.2 wt % NaCl at a concentration of 5 mg/mL. To convert GAG carboxylic acid groups to thiols, dithio-bis(propionohydrazide) (DTP) was added in sufficient quantity to convert 18% of the GAG carboxylic acid groups. Conjugation was performed using 1-ethyl-3-(3-(dimethylamino)propyl)carbodiimide (EDC), which was added in a 2:1 ratio of EDC to DTP. The solutions were titrated to pH 4.5 and reacted overnight. The disulfide bonds of DTP were cleaved by titrating the solutions to pH 8.5, and dithiothreitol (DTT) was added in a 3:1 molar excess of DTP. After 3 h at room temperature, the solutions were titrated to pH 4.5 to prevent the reformation of disulfide bonds. The polymer solutions were then purified using a KrossFlo KR2i tangential flow filtration (TFF) unit (Repligen) with a 10 kDa molecular weight cut-off column and a transmembrane pressure of 18 PSI. The polymer solutions were purified until a permeate volume three times the reaction volume was reached. Following purification, the polymer solutions were filtered through a 0.2 μm filter, frozen, and lyophilized. Free thiol content of the thiolated GAGs was determined using Ellman’s assay, with the degree of thiolation defined as the percentage of GAG carboxylic acid groups converted to free thiols (Figure S1 and Table S1).

### 2.2 Fabrication of Blended GAG Hydrogels and GAG/Collagen IPNs

The dry polymer was first sterilized by immersion in absolute ethanol. Excess ethanol was removed, and the polymer was dried in a laminar flow hood. Stock solutions of HA-SH and CS-SH were prepared by dissolving GAGs in phosphate-buffered saline (PBS) at a concentration of 80.8 mg/mL. To prepare blended GAG hydrogels, stock GAG solutions were made of 7:3 CS-SH to HA-SH or 3:7 CS-SH to HA-SH. GAG hydrogels at 3 wt % were prepared by combining 150 μL of 20 mM acetic acid, 35 μL of PBS, 25 μL of 260 mg/mL poly(ethylene glycol) diacrylate (PEGDA), and 130 μL of the blended GAG solutions. The pregel solutions were titrated to a pH of 7.8. For GAG/collagen IPNs, 20 mM acetic acid solution was substituted by a 9.33 mg/mL solution of rat tail collagen type I (Corning), leading to a final collagen concentration of 0.4 wt %. Following titration, 90 μL of gels were made by placing the solution into silicone molds (8 mm in diameter, 1 mm in thickness) and incubating them at 37 °C for 2 h in a humidified environment. Collagen-only gels were made by mixing 265 μL of 9.33 mg/mL collagen type I solution with 35 μL of 10× PBS, titrating to pH 7.8, and then mixing in 35 μL of PBS. Collagen gels were pipetted into the same silicone molds, which mimic the thickness of native cartilage, and then incubated at 37 °C for 2 h.^44^

### 2.3 Mechanical Testing of Hydrogels

Compression mechanical testing was performed by using a Discovery Hybrid Rheometer (TA Instruments). Prior to compression testing, hydrogels were allowed to swell overnight in PBS at 37 °C in a humidified environment. Hydrogels were placed on the stage under an 8 mm head and compressed until 50% strain was reached at a constant rate of 5 μm/s. The compressive modulus was then calculated from the linear portion of the stress/strain curve.

### 2.4 Characterization of Hydrogel Swelling and Diffusive Properties

For the characterization of hydrogel swelling as a function of composition, gels were made directly in 0.5 mL centrifuge tubes. Following gelation, PBS was pipetted atop, and the tubes were allowed to equilibrate. After swelling, a new mass of the hydrogel was obtained, and the hydrogel was washed three times with deionized water to remove the salts. The hydrogels were then lyophilized, and the swelling ratio was calculated as the ratio between the swollen and dry masses.

To characterize the diffusivity of the hydrogels, they were loaded with rhodamine isothiocyanate labeled 70 kDa dextran (dextran-RITC) prior to gelation. Following gelation, the gels were submerged in PBS, and the release of dextran-RITC from the hydrogels was determined from the fluorescence of the supernatant. Dextran-RITC release from the hydrogels was monitored over the course of 1 week.

To characterize the binding capacity of IL-1β to the different gel formulations, acellular gels were placed in 350 μL of Dulbecco’s modified Eagle’s medium (DMEM) (Gibco), supplemented with 10% fetal bovine serum (FBS), 1% penicillin/streptomycin/amphotericin (Gibco), and 20 ng/mL recombinant human IL-1β (Peprotech). After 2 days of immersion, the media were collected and analyzed for IL-1β content using a human-specific IL-1β enzyme-linked immunosorbent assay (ELISA) kit (R&D systems). The binding capacity of the IL-1β to gels was determined by calculating IL-1β depletion from the media, which was calculated based on the difference between the initial and final concentrations of IL-1β in the media.

### 2.5 Primary Animal Chondrocyte Isolation and Culture

The primary fetal bovine articular chondrocytes (fbACs) were isolated from the cartilage of the hind knees of fetal bovines (Animal Technologies) according to previously reported methods.^45−48^ Cartilage slices were shaved off the medial condyle and digested in a solution of 0.2 w/v% collagenase P (Millipore Sigma), 0.1 w/v% bovine serum albumin (Millipore Sigma), and 3% FBS (Gibco) for 2 h. Following digestion, the cells were strained out of the undigested cartilage through a 70 μm cell strainer. The collected cells were either cryopreserved in Cryo-SFM (Promocell) until needed or expanded in DMEM (Gibco) supplemented with 10% FBS and 1% penicillin/streptomycin/amphotericin (Gibco). Cells were used between passages 2 and 5. To encapsulate fbACs in hydrogels, 35 μL of PBS was substituted with 35 μL of PBS with 20 × 10^6^ cells/mL for a final cell concentration of 2 × 10^6^ cells/mL.

### 2.6 Chondrocyte Culture in Pro-inflammatory Conditions

#### 2.6.1 Effect of Pro-Inflammatory Conditions on Hydrogels

Cell-laden hydrogels were either cultured in pro-inflammatory media, which consisted of the cell expansion medium supplemented with 50 μg/mL ascorbic acid (Millipore Sigma) and 20 ng/mL recombinant human IL-1β (Peprotech) to simulate the osteoarthritic environment,^49^ or in noninflammatory media, which consisted of the cell expansion medium supplemented with 50 μg/mL ascorbic acid and 40 ng/mL dexamethasone (Millipore Sigma).^50^ Hydrogels were cultured in 48-well plates with media exchanged and collected every 2 days. fbACs were encapsulated at a final cell concentration of 2 million cells/mL.

#### 2.6.2 Analysis of Cellular DNA and Metabolic Changes in Response to Pro-inflammatory Conditions

To measure changes in hydrogel DNA content following pro-inflammatory culture, hydrogels were placed in 400 μL of 1 mg/mL hyaluronidase type I–S (Millipore Sigma) and 1× proteinase inhibitor (ThermoFisher), mechanically homogenized with a rotary tissue homogenizer, and digested overnight. Next, 50 μL of the gel digest was combined with 50 μL of PicoGreen reagent (ThermoFisher), and DNA concentration was determined using the fluorescent signal at excitation and emission wavelengths of 485 nm and 535 nm, respectively. A DNA calibration curve was constructed by using lambda phage DNA (ThermoFisher).

Changes in the fbAC metabolic rate as a result of IL-1β stimulation were determined using AlamarBlue (Invitrogen), according to the manufacturer’s instructions. All cell-laden hydrogels were cultured in AlamarBlue-containing media with and without IL-1β for 4 h. The cells were examined 1 day after encapsulation and again after a 14-day culture period. The fluorescence signal of the medium after the culture period was normalized to the initial time point to determine the changes in cell metabolism.

#### 2.6.3 Effect of Pro-inflammatory Conditions on Hydrogel Integrity

To assess how the pro-inflammatory conditions affected the physical properties of the cell-laden material, changes in wet and dry masses, as well as changes to compressive strength, were measured. After 7 and 14 days, the hydrogels were harvested and weighed in preweighed centrifuge tubes. The compressive strength was then tested using the methods previously described. Finally, the hydrogels were frozen, lyophilized, and weighed again to determine their dry mass. Furthermore, the amount of CS released into the medium during culture was determined by using a dimethylmethylene blue (DMMB) assay.

#### 2.6.4 Analysis of Secreted Cytokines in Response to Pro-inflammatory Conditions

To determine the ability of IL-1β to induce inflammation in the embedded chondrocytes, the secretion of interleukin 6 (IL-6), interleukin 8 (IL-8), chemokine ligand 2 (CCL2), and MMP13 as markers of inflammation was analyzed. During culture in the pro-inflammatory media, the media were collected every 2 days, and the collected media were pooled together and frozen at −80 °C until analysis. IL-6 production was measured using a bovine-specific IL-6 ELISA kit (R&D Systems). IL-8 production was measured using a bovine-specific IL-8 ELISA kit (Mabtech). CCL2 production was measured using a bovine-specific CCL2 ELISA kit (Kingfisher Biotech). MMP13 production was determined based on the MMP13 activity of the media and measured using an MMP13 fluorometric activity assay (Anaspec). All kits were used according to the manufacturer’s specifications.

#### 2.6.5 Analysis of Gene Expression in Response to Pro-inflammatory Conditions

To determine the effects of IL-1β on the expression of anabolic and catabolic chondrocyte genes, the mRNA of the embedded fbACs was analyzed using quantitative polymerase chain reaction (qPCR) after 14 days of either pro-inflammatory or noninflammatory culture. Following culture, the hydrogels were immersed in TRIzol LS according to the manufacturer’s directions and then homogenized using a Tissue Tearor rotor homogenizer. Samples were centrifuged to remove solid components, and mRNA was extracted from the supernatant according to the manufacturer’s instructions. Following mRNA extraction, cDNA was synthesized using a High-Capacity cDNA Reverse Transcription kit (Invitrogen), according to the manufacturer’s instructions.

qPCR was performed using Taqman probes (Thermofisher) targeting collagen II and aggrecan as markers of chondrogenic anabolic activity, collagen I, and collagen X as markers of hypertrophy, and MMP13 and ADAMTS5 as markers of inflammation-induced catabolic activity. Regulation of gene expression was analyzed using the ΔΔ*C*_t_ method, with expression of genes of interest first normalized to the expression of GAPDH and then to either the IL-1β stimulated collagen-only hydrogel control or to each group’s respective unstimulated control.

#### 2.6.6 Immunohistochemical Analysis of Gels Cultured in Pro-inflammatory Conditions

To visualize the production of the chondrogenic markers aggrecan and collagen, gels cultured under pro-inflammatory conditions were sectioned, stained, and analyzed using immunohistochemistry. Immediately after 2 weeks of culture, the gels were fixed with 4% paraformaldehyde in PBS for 30 min. Following fixation, the hydrogels were embedded in 3% agarose solution in 15 mm × 15 mm × 5 m plastic base molds (Electron Microscopy Sciences 6235215). Using a vibratome (Leica VT1200), a 300 μm thick slice was obtained. Slices were placed in 24-well glass-bottom plates (Cellvis, P24-1.5H-N). The samples were permeabilized with 1% Triton X100 for 15 min and washed with 1% BSA in 1× PBS. The gels were then blocked with 200 μL of 3% BSA in 1× PBS for 1 h. The gels were rinsed in 1% BSA in 1× PBS three times for 5 min each. The samples were stained with Hoechst dye 33258 at 2 μg/mL in 1× PBS for 10 min. The samples were washed in 1% BSA in 1× PBS three times for 5 min. Then, 200 μL solutions of rabbit anticollagen II antibody (ab34712, Abcam, Cambridge, MA) diluted 1:200 and mouse antiaggrecan (ab3778, Abcam, Cambridge, MA) diluted 1:100 in 1% BSA in 1× PBS were incubated with each gel slice overnight at 4 °C. Gels were rinsed in 1% BSA in 1× PBS for 5, 10, and 15 min. A 200 μL solution of donkey antimouse IgG Alexa Fluor 546 (A10036, Invitrogen, Carlsbad, CA) diluted 1:200 and a solution of goat antirabbit IgG Alexa Fluor 488 (A11008, Invitrogen, Carlsbad, CA) in 1% BSA in 1× PBS were incubated with the gel slices overnight at 4 °C. The gels were rinsed with 1% BSA in 1× PBS for 5, 10, and 15 min, and immediately imaged using an LSM 900 confocal microscope (Zeiss, Jena, Germany). The samples were imaged using 10× and 20× objectives (Zeiss Plan-Apochromat 10×/0.45 M27 and 20×/0.8 M27). Images of size 512 × 512 pixels were captured at three different horizontal locations per gel, and three replicates of each gel group were imaged, for a total of *n* = 9 images per gel group. Z-stacks were taken at 20 μm intervals unless otherwise stated, through the hydrogels and processed using maximum projection. Quantification of aggrecan and collagen signals was performed using ImageJ, by determining the average pixel intensity and area of aggrecan and collagen signals and normalizing to the area of the cell nuclei stained with DAPI.

### 2.7 Statistical Analysis

Experiments involving the physical characterization and biochemical analysis of the cultured hydrogels were performed in replicates of six. Experiments involving qPCR and immunohistochemical staining of the hydrogels were performed in triplicates. Statistical significance between the two groups was determined using Student’s *t*-test. For comparisons between the stimulated and unstimulated groups, multiple *t*-tests were performed, one for each gel type. Statistical significance between three or more groups was determined using a one-way ANOVA, with significance between groups determined using a Tukey post hoc test. Statistical analysis was done with GraphPad Prism software. A probability value of 95% (*P* < 0.05) was used to determine statistical significance.

## 3 Results

### 3.1 Effect of GAG Ratio and the Inclusion of Collagen on Hydrogel Physical Properties

**Figure 1:**
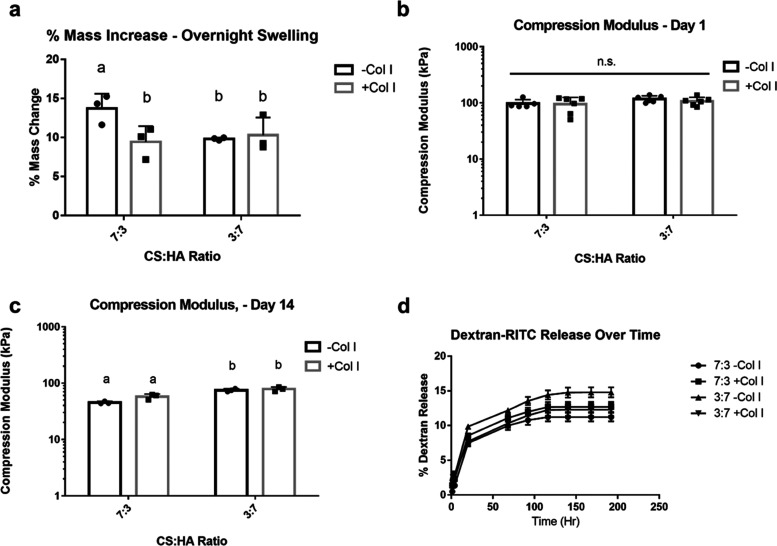
Physical properties of GAG hydrogels with different GAG blends and collagen contents. (A) Change in mass of hydrogels (%) following overnight swelling in PBS. (B) Initial compression modulus of the swollen hydrogels. (C) Compression modulus after 14 days of acellular culture. (D) Release profiles of dextran-RITC over 196 h. The groups sharing the same letter are statistically similar to one another (*P* > 0.05). The groups denoted by n.s. are not statistically significant from one another.

For all studies, hydrogels fabricated from a blend of CS and HA were used based on previous studies that demonstrated superior qualities over homopolymer hydrogels, including promoting cell viability and supporting resistance to enzymatic degradation.^42^ One consequence of modulating the ratio of CS to HA in the blended GAG hydrogels was a change in the charge density of the hydrogel due to the difference in charge between the sulfated CS and unsulfated HA. As a result, the 7:3 CS/HA hydrogel exhibited increased swelling compared to the 3:7 CS/HA hydrogel, likely due to the increased negative charge of the 7:3 CS/HA hydrogel (Figure 1a). However, the inclusion of collagen in the 7:3 hydrogel reduced hydrogel swelling, making it equal to that of the 3:7 hydrogel with and without collagen. This reduction may be due to the stiffness of collagen networks, which conferred resistance to swelling in the 7:3 formulation. Although collagen did not decrease swelling in the 3:7 formulation, this is likely due to the decreased potential for swelling in HA-dominant gels, as previously described.^42^ Despite differences in swelling, the initial compressive moduli of all hydrogel formulations, with and without collagen, in their swollen state were equivalent (Figure 1b, Table 1). However, after 2 weeks of incubation in cell media, the acellular hydrogels had reduced compressive strength (Figure 1c, Table 1). This was most apparent in the 7:3 formulation without collagen hydrogel, which lost nearly half of its initial compressive modulus.

**Table 1:** Initial and Final Compressive Moduli of CS/HA Blended Hydrogels

	initial compressive modulus	final compressive modulus
7:3 CS/HA – Collagen I	96.9 ± 15.2 kPa	45.1 ± 1.8 kPa
7:3 CS/HA + Collagen I	94.6 ± 27.8 kPa	57.7 ± 5.2 kPa
3:7 CS/HA – Collagen I	117.8 ± 13.9 kPa	74.8 ± 3.6 kPa
3:7 CS/HA + Collagen I	107.0 ± 16.7 kPa	78 ± 5.5 kPa

To characterize how the hydrogel formulation affected solute diffusivity, particularly macromolecule diffusion, hydrogels were loaded with dextran-RITC and the mass of dextran-RITC released was monitored for 1 week (Figure 1d). Over the course of 1 week, the long-term release profiles of the 70 kDa dextran-RITC from the different GAG gel formulations were similar, suggesting equivalent diffusivities despite changes in the CS/HA ratio and the inclusion of collagen.

We evaluated the potential of the inflammatory cytokine IL-1β, which is present in the fluid surrounding the hydrogel, to bind to and potentially be sequestered by the hydrogel. Although the 7:3 gels contained more CS, which could have led to a more negatively charged hydrogel surface, there was no significant difference in IL-1β binding between any of the GAG gel groups, regardless of the inclusion of collagen (Figure S2). This could be due to the already high CS content in all GAG hydrogel groups, with an increase from 0.9 to 2.1 wt % CS having very little effect on the concentration of IL-1β investigated. However, all GAG hydrogels demonstrated increased IL-1β binding compared to the collagen-only gel, suggesting increased interaction with the material due to the presence of GAG within the gel (Figure S2).

### 3.2 Changes in Hydrogel Integrity Following Culture in Pro-Inflammatory Conditions

**Figure 2:**
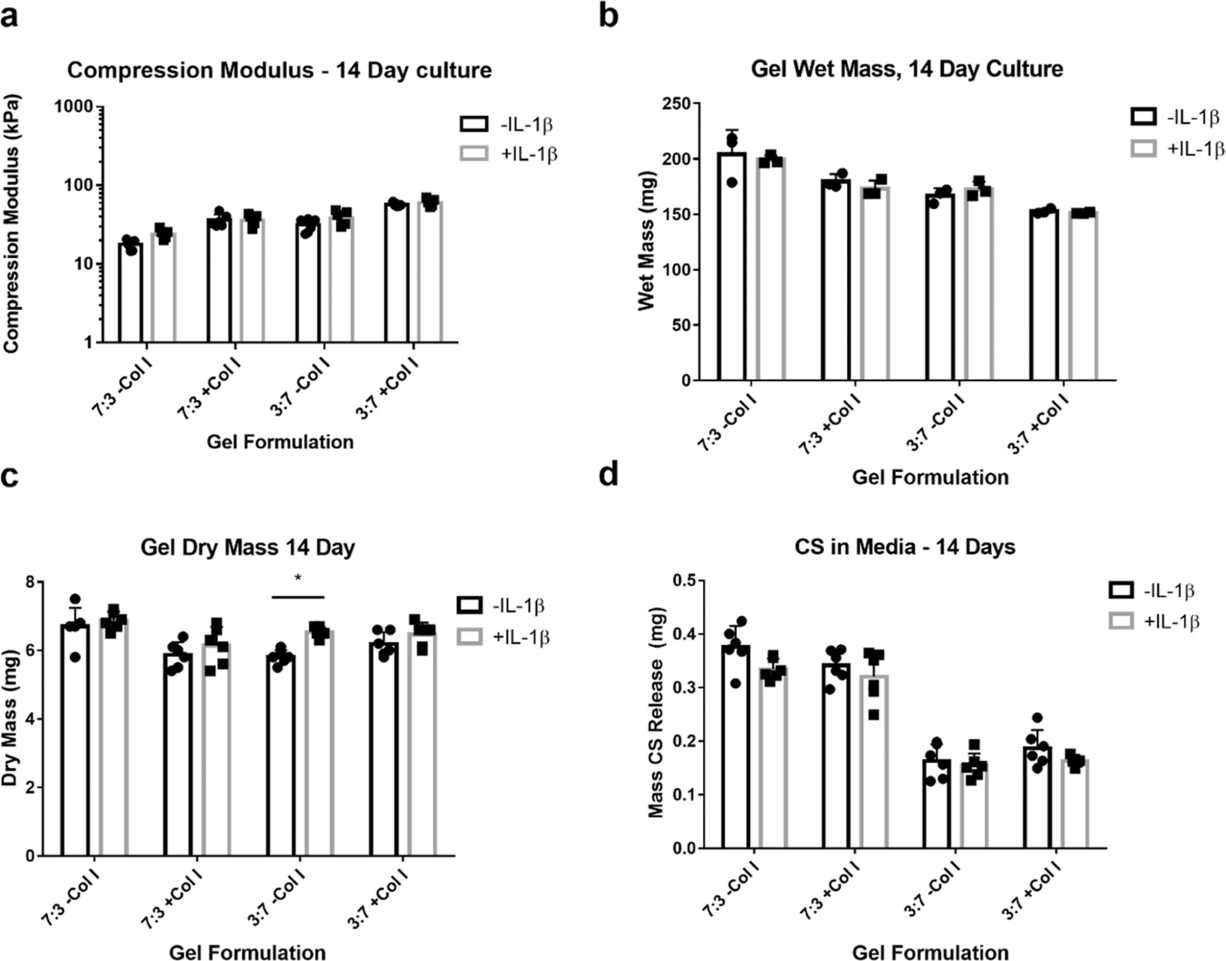
Changes in fbAC-laden GAG hydrogel integrity over 14 days in IL-1β-stimulated and -unstimulated cultures. (A) Compression moduli of GAG gels, (B) gel wet mass, (C) gel dry mass, and (D) cumulative CS release into media over 14 days of culture. Multiple *t-*tests were performed to compare the stimulated and unstimulated groups. Groups marked with * show significant differences between groups (*P* < 0.05).

To understand the changes in the encapsulated cell behavior in response to their environment when cultured in pro-inflammatory conditions, changes in hydrogel mass and compressive strength were tested. During 14 days of culture, all cell-laden hydrogels exhibited a decrease in compressive strength (Figure 2a). The degree of loss of compressive strength correlated with the formulation of the hydrogel rather than the media formulation. Similar to the acellular hydrogels, the formulations exhibiting the largest decrease in strength were the 7:3 groups. The addition of collagen to both GAG blends reduced the loss of stiffness. Furthermore, all groups exhibited a compressive strength lower than that of their acellular counterparts, indicating that this change was due in part to cell activity. However, there were no significant differences in the compressive strength between the hydrogels cultured in the pro-inflammatory and the noninflammatory media, suggesting that this behavior was due to normal cell remodeling and not due to inflammatory stimulation. Further measurements of the wet (Figure 2b) and dry masses (Figure 2c) of the hydrogels, as well as the CS released into the media (Figure 2d), showed no significant differences between hydrogels cultured in pro- and noninflammatory conditions, with the exception of the 3:7 CS/HA-Col I group, which showed a small but significant difference in the final dry mass between the groups stimulated with and without IL-1β (Figure 2c). Overall, stimulating the constructs with 20 ng/mL IL-1β had no large effects on hydrogel integrity for the majority of gel formulations, indicating that the encapsulated cells did not significantly degrade the scaffold under pro-inflammatory conditions.

### 3.3 Changes in Cell Behavior under Pro-Inflammatory Conditions

**Figure 3:**
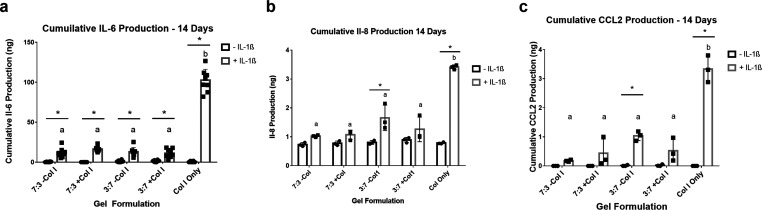
Changes in fbAC-soluble factor secretion following IL-1β stimulation. (A) Cumulative IL-6 production, (B) cumulative IL-8 production, and (C) cumulative CCL2 production after 14 days of culture. * denotes statistical significance (*P* < 0.05) between gels cultured with and without IL-1β. Letters (a and b) denote statistical significance between groups cultured with IL-1β. Groups that do not share letters are statistically significantly different (*P* < 0.05) from the other groups.

To quantify the inflammatory response and catabolic activity of the encapsulated fbACs, the media of the cultured constructs were investigated for the presence of IL-6, IL-8, CCL2, and MMP13. When subjected to pro-inflammatory conditions, the secretion of these factors is upregulated by chondrocytes.^30,36,51−53^ As such, the increased production of these factors was inferred to equate to an upregulation of pro-inflammatory behavior in response to IL-1β. All groups stimulated with IL-1β produced significantly more IL-6 than their respective unstimulated counterparts (Figure 3a). However, with regard to IL-8 and CCL2, only the 3:7 –Col I and collagen-only groups saw significant increases in cytokine secretion (Figure 3b,c). In the remaining groups, there was a nonsignificant but trending increase in the production of these factors (*P* > 0.05).

When comparing the groups stimulated with IL-1β, the fbACs encapsulated in the collagen-only gel produced significantly more IL-6 (Figure 3a), IL-8 (Figure 3b), CCL2 (Figure 3c), and MMP13 (Figure S3). Among GAG-containing gels, there were no significant differences in the production of these factors. In the cases of IL-8 and CCL2, the 3:7 –Col I group tended to have higher levels than the other groups, although this increase was not significant. Similarly, MMP13 production was higher in the groups that were predominantly HA than in the groups that were predominantly CS; however, this difference was not significant.

In addition to the secretion of pro-inflammatory cytokines, another marker of inflammation that was measured was the change in cell number under pro-inflammatory conditions. Changes in cell numbers were measured indirectly by measuring the DNA content of the hydrogels following culture. In the GAG hydrogels, there was no significant change in DNA content between gels cultured in standard and pro-inflammatory conditions (Figure S4). In contrast, collagen gels cultured under pro-inflammatory conditions contained significantly more DNA than those cultured under normal conditions (Figure S4). Similar trends were seen with regard to the chondrocyte metabolic rate for cells encapsulated in GAG gels (Figure S5).

### 3.4 Changes in Expression of Chondrogenic Genes

To further quantify the effect of culture in pro-inflammatory conditions on the encapsulated fbACs, isolated mRNA was analyzed using qPCR to examine changes in gene expression. We normalized the changes in gene expression in two different ways to facilitate comparison. First, to assess the effect of IL-1β on the encapsulated cells given a certain scaffold formulation, we normalized the GAG gels treated with IL-1β to the corresponding unstimulated GAG gel. Second, to assess how culture in GAG blend gels under pro-inflammatory conditions differed from the collagen gel control, we normalized gel groups stimulated with IL-1β to the collagen-only gel stimulated with IL-1β.

**Figure 4:**
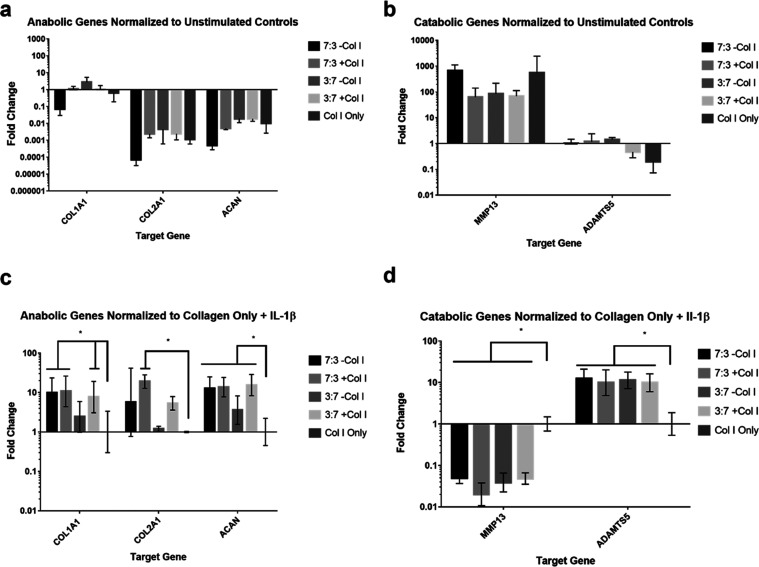
Changes in gene expression after 14 days of IL-1β-stimulated culture. Changes in (A) anabolic and (B) catabolic gene expression normalized to the respective unstimulated groups. Changes in (C) anabolic and (D) catabolic gene expression normalized to IL-1β-stimulated collagen-only gel.* Significant differences between groups (*P* < 0.05).

In general, when comparing all GAG hydrogel groups stimulated with IL-1β to their unstimulated counterparts, all cartilage matrix-related genes were downregulated and MMP13 was upregulated (Figure 4a,b). There were no significant changes in the expression of collagen type I and ADAMTS5 in response to IL-1β stimulation. The expression of collagen type X was not detected in any of the groups, regardless of IL-1β stimulation. While the downregulation of chondrogenic genes suggests that the cells of all groups are affected by IL-1β, a lack of change in collagen type I and X indicates that IL-1β does not contribute to any hypertrophic differentiation of chondrocytes.

When normalized to the collagen-only gel control stimulated with IL-1β, all groups showed a decrease in MMP13 expression and an increase in collagen type II and aggrecan expression (Figure 4c,d). Collagen type I and ADAMTS5 showed increased expression compared to the collagen-only gel, and the lack of change compared to the unstimulated controls suggests that this increase in expression may be due to the gel composition rather than the inflammatory effects of IL-1β. Overall, the culture in the GAG gels under pro-inflammatory conditions promoted the expression of certain chondrogenic genes, although the expression of these genes was still lower than that observed in the respective unstimulated controls.

### 3.5 Visualization and Quantification of Chondrogenic Markers Produced under Pro-inflammatory Conditions

**Figure 5:**
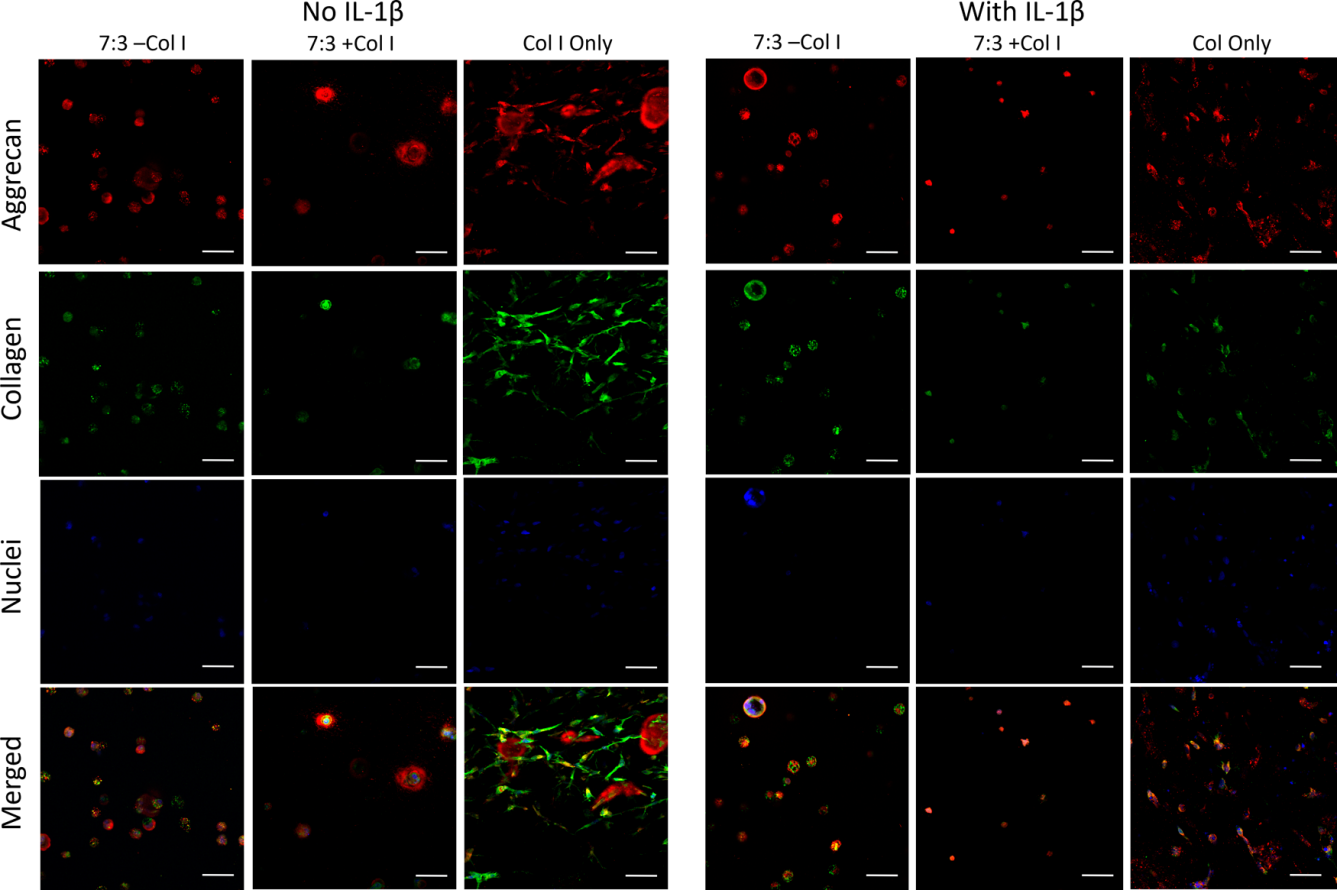
Immunohistochemical staining of aggrecan and collagen produced by fbACs cultured in GAG and collagen gels with and without IL-1β. Images are composed of a maximum projection *Z*-stack of a 300 μm-thick sample section with images taken every 20 μm. The scale bar represents 50 μm.

To visualize the production of the cartilage ECM proteins aggrecan and collagen, cell-laden gels cultured under standard and pro-inflammatory conditions were sectioned and subsequently stained with antibodies targeting these proteins, and the nuclei of fbACs were visualized using DAPI. The fbACs encapsulated in GAG gels remained dispersed, whereas the fbACs in collagen gels appeared closer together due to the compaction of the collagen gels by the encapsulated cells (Figures 5 and S6). For cells encapsulated in GAG gels and cultured under noninflammatory conditions, all imaged cells displayed localized production of aggrecan and collagen, as denoted by the colocalization of the visualized aggrecan and collagen around the cell nuclei (Figures 5 and S6) In contrast, while many of the cells cultured in the collagen gels produced collagen, aggrecan production was most apparent in the cells that were clustered together (Figures 5 and S6).

**Figure 6:**
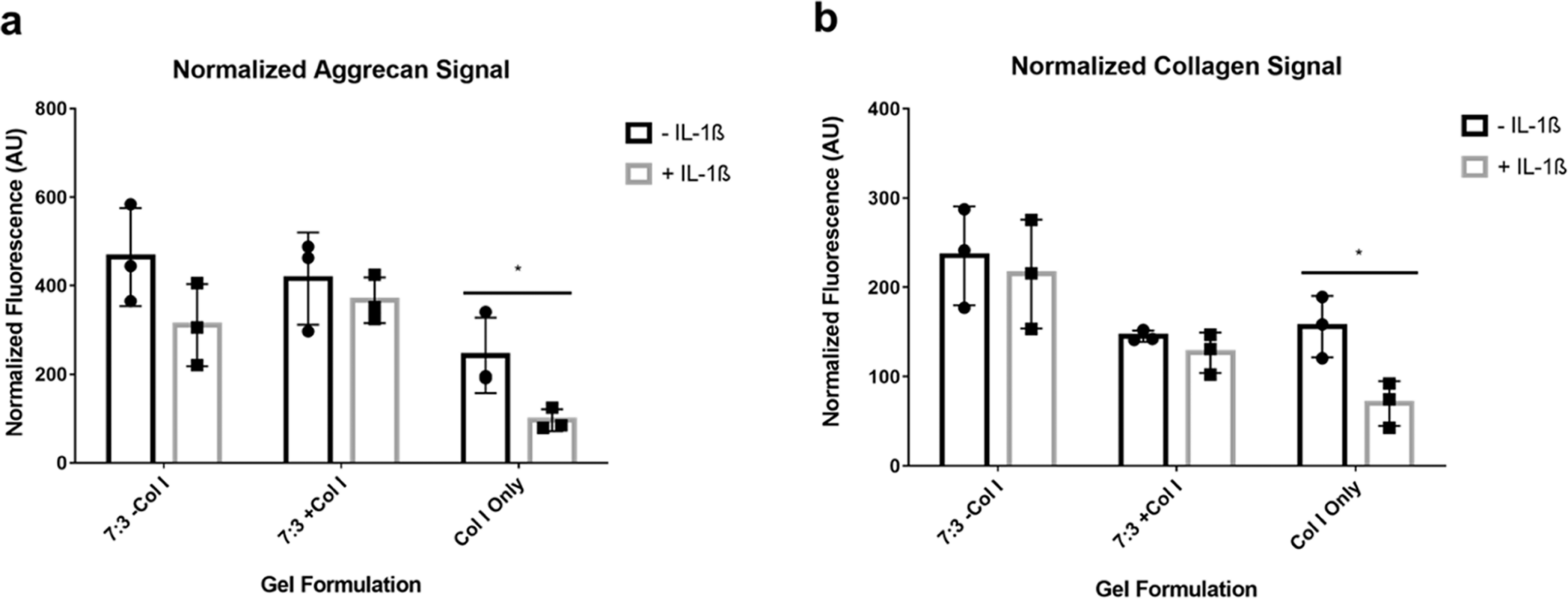
Quantification of immunohistochemical staining of aggrecan and collagen produced by fbACs cultured in GAG and collagen gels with and without IL-1β. * Statistical significance (*P* < 0.05) between gels cultured with and without IL-1β.

When compared with cells cultured in pro-inflammatory conditions, similar amounts of aggrecan and collagen were detected in GAG gels (Figures 6 and S7). Again, in the GAG gels, aggrecan and collagen were detected colocalized with cells, as indicated by proximity to their DAPI nuclei signals. Morphological differences were detected in the fbACs cultured in GAG gels containing collagen (7:3 + Col, 3:7 + Col), with the size of the cells smaller on average compared to their unstimulated counterparts. In contrast, cells cultured in GAG gels without collagen (7:3 −Col, 3:7 −Col) maintained similar cell sizes. However, aggrecan and collagen were still detected close to the cell nuclei. Quantification of aggrecan and collagen fluorescence in the unstimulated and stimulated groups of GAG gels showed similar levels of both proteins (Figures 6 and S7), suggesting preservation of aggrecan and collagen production during culture in pro-inflammatory conditions.

In the collagen-only gels, the observed nuclear density is higher than expected (Figure 5) based on the 2–3-fold increase in DNA compared to the gels containing GAG (Figure S3). This is consistent with the observed compaction of the collagen-only gels, which did not occur to an appreciable extent in GAG-containing gels. The DNA content and high compaction of the gels should be taken into account when considering aggrecan and collagen staining. Although collagen and aggrecan densities appear higher in collagen-only gels, this is due in part to the high compaction of the gels, which increases the local density of the matrix. When normalized to the cell nuclear fluorescence signal, unstimulated gels had similar levels of aggrecan and collagen (Figures 6 and S7). However, in contrast with the GAG gels, the stimulated collagen-only gel showed a significant decrease in both aggrecan and collagen compared to the unstimulated collagen-only gel (Figure 6), suggesting a susceptibility to pro-inflammatory cytokines consistent with other findings.

## 4 Discussion

Although OA has traditionally been considered a cartilage disease resulting from mechanical wear of the articular cartilage, recent studies have shown that inflammation is a major component of OA pathophysiology. As such, tissue engineering strategies that seek to replace cartilage defects that result from OA should consider the pro-inflammatory environment in which they will be implanted. While many studies have demonstrated the ability to promote cartilage matrix production and others have demonstrated methods to attenuate chondrocyte inflammation, few have studied the engineering of cartilage in an inflammatory environment.

In this study, we developed hydrogels composed of CS and HA to take advantage of their anti-inflammatory and chondrogenic properties. Furthermore, CS and HA were minimally modified to maximize the biological signals present in the encapsulated cells.^42^ In addition to GAGs, collagen type I was added to some of the gels to incorporate additional cell-binding sites and to provide elastance to counteract the swelling of the GAG gels. Using two ratios of CS and HA plus collagen, we formulated four hydrogels that had similar compressive strength and diffusivity but presented different biological signals to the encapsulated cells. Collagen-only gels served as a control and as an analogue to the collagen-based scaffolds used in OA treatments such as ACI.^54^

One hallmark of OA is an increase in catabolic activity, resulting in the enzymatic breakdown of articular cartilage. Here, there were no significant increases in the degradation in the GAG hydrogels following 14 days of stimulation with IL-1β, as determined by the lack of mass change and stable compressive strength, compared to unstimulated matched constructs. At the same time, all cell-laden gels decreased in compressive strength over the culture period to a similar extent but to a greater extent than the acellular hydrogels over the same period. This data suggests that changes in hydrogel integrity may be due to cellular activity that is not associated with inflammation. This is further supported by the lack of differences in hydrogel mass and release of CS into the media, with the majority of gel formulations being between the stimulated and unstimulated controls. Overall, this suggests that chondrocytes encapsulated in these GAG blend gels do not significantly degrade the hydrogels in response to pro-inflammatory signals. The exception was the difference in the dry mass of the gels with the 3:7 −Col formulation, where a small but significant change in mass occurred. Under pro-inflammatory conditions, it has been demonstrated that chondrocyte hyaluronidase activity is upregulated.^55−60^ In OA, this upregulation of hyaluronidase activity is responsible for the fragmentation of HA and the release of aggrecan from the cartilage. Hyaluronidase can be both cell-membrane-bound^58,59^ and extracellular.^56^ Since we have previously shown that both our thiolated HA and thiolated CS are susceptible to hyaluronidase activity,^42^ and the data presented here shows little to no GAG degradation, it is likely that the hydrogel environment inhibits the increased expression of soluble and membrane-bound hyaluronidases through anti-inflammatory activity.

Another hallmark of OA is the secretion of inflammation-related soluble factors including cytokines, chemokines, and enzymes. To quantify the changes in inflammation-related factor secretion, we chose to quantify several molecules implicated in a variety of pro-inflammatory processes, including IL-6,^51,61^ IL-8,^62^ CCL2,^63−65^ and MMP13^66^ when constructs were treated with IL-1β. Comparing stimulated and unstimulated gels, a significant difference in secretion within all-gel groups was observed with regard to IL-6. However, with Il-8 and CCL2, significant increases in secretion compared to the unstimulated baseline were observed only in the 3:7 −Col and Col-only groups, demonstrating that these groups were more responsive to inflammation. From these data, it can be inferred that regardless of the formulation, all groups responded to the pro-inflammatory stimulus to some degree. These differences may be explained by differences in cytokine/GAG interactions with cytokines that potentially bind to CS better than to HA, resulting in sequestration from cells. Furthermore, when comparing IL-1β-stimulated GAG-containing gels to collagen-only gels, the presence of GAG significantly attenuated the pro-inflammatory response measured by IL-6, IL-8, CCL2, and MMP13 secretion. Overall, GAG hydrogels appear to offer protection to the encapsulated cells from external pro-inflammatory stimuli, with the ratio of CS/HA and the inclusion of collagen in the GAG network potentially having an additional effect, and this protection is noticeably better than that of a control collagen hydrogel.

In addition to soluble factor secretion, the change in cell number was used as an indirect measure of changes in cell proliferation or death under pro-inflammatory conditions. DNA content isolated from the hydrogels was used as a proxy for cell number. For the GAG hydrogels, there were no significant differences in the DNA content between the unstimulated and IL-1β-stimulated groups. However, in the collagen-only hydrogel groups, stimulation with IL-1β for 14 days of culture resulted in a significant increase in the DNA content measured. While it is generally believed that stimulation of articular chondrocytes with pro-inflammatory cytokines results in cell apoptosis,^67^ stimulation of juvenile chondrocytes has been found to result in increased proliferation.^68−70^ Given the fetal nature of the chondrocytes used in these experiments, the increase in DNA seen in the IL-1β-stimulated collagen-only gel groups would align with these findings in juvenile cells, further suggesting that IL-1β was able to stimulate the fbACs more readily in the collagen-only gel than in the GAG gels.

As an assessment of catabolic activity, we measured the gene expression of MMP-13 and ADAMTS5. Two points of comparison were studied to better understand the protective effects of the GAG gels from the pro-inflammatory environment: how gene expression changed compared to an IL-1β-stimulated collagen type I gel and how gene expression changed compared to each group’s respective unstimulated control. When compared to the collagen type I gel, all GAG gels showed decreased expression of MMP13 but increased expression of ADAMTS5. However, when compared to their respective unstimulated gels, there was no significant change in ADAMTS5 expression, suggesting that increased ADAMTS5 expression was due to culture in a GAG gel versus a collagen-only gel and was unrelated to the inflammatory environment. The lack of change in ADAMTS5 expression may be explained by the choice of pro-inflammatory cytokines in this model. In OA, increases in ADAMTS5 expression have been correlated with increased TNF-α secretion,^71^ whereas IL-1β stimulates ADAMTS4 production.^72^ Increased expression of MMP13 was observed in the IL-1β-stimulated GAG gels, suggesting that although chondrocytes in these gels expressed MMP13 to a lesser degree than those in the stimulated collagen gel, the expression was higher than that observed in unstimulated controls. Similar to the previously detailed markers of inflammation, the downregulation of MMP13 compared to the collagen-only gel but upregulation compared to the unstimulated controls suggests a dampening response to inflammation but not complete protection from it.

To examine the preservation of chondrogenic activity while under pro-inflammatory conditions, we examined the gene expression of cartilage matrix proteins collagen type II and aggrecan. Following 14 days of culture, the gene expression of collagen type II and aggrecan increased, as did collagen type I in all GAG gel groups, with the exception of the 3:7 CS/HA without the collagen group. Similar to the catabolic genes, while there was an increase in the expression of these genes over the collagen type I gel, the expression of collagen type II and aggrecan was still downregulated when compared to their expression in their respective unstimulated controls. Furthermore, the expression of collagen type I did not change significantly between the stimulated gels and their unstimulated controls, again suggesting that collagen type I was not upregulated by IL-1β stimulation.

Finally, the production of cartilage ECM proteins aggrecan and collagen was confirmed and quantified by using immunohistochemistry. When comparing the unstimulated and pro-inflammatory-stimulated groups, the fbACs encapsulated in GAG hydrogels produced similar levels of aggrecan and collagen, with all detected cell nuclei colocalized with aggrecan and collagen. Additionally, morphological differences were observed in the GAG gels that also contained collagen, with fewer clusters of cells observed, and a reduced average cell size was seen in the IL-1β-stimulated GAG + Col gels. In contrast, fbACs in GAG-only gels maintained similar morphologies. However, for collagen-only gels, a significant difference in aggrecan and collagen production was observed. While collagen was observed in most cells and aggrecan was seen with certain cell clusters in the unstimulated gels, reduced production of both was present in the IL-1β-stimulated gels. In all GAG groups and the unstimulated collagen gels, aggrecan and collagen were seen colocalized to nearly all visualized nuclei, and a large number of cell nuclei without aggrecan and collagen were detected in the IL-1β-stimulated collagen gel groups. When controlling for cell nuclei, cells in collagen-only gels exhibited a significant decrease in the levels of aggrecan and collagen production when comparing the stimulated and unstimulated groups. In contrast, the differences between the IL-1β-stimulated and -unstimulated GAG gels were insignificant, with only a decreasing trend between cells in pro-inflammatory and standard environments. These findings are consistent with the changes in aggrecan and collagen gene expression, which were also downregulated compared to the majority of GAG hydrogel groups as well as the unstimulated collagen gel group.

Overall, the blended GAG hydrogels were able to partially protect against inflammation from relatively high and sustained doses of IL-1β in the culture media over the course of 14 days.^29,37,53,73^ While minor differences were seen between formulations, with the exception of the 3:7 −Col group, all GAG gel groups protected from inflammation more readily than the collagen-only gel. Furthermore, this was accomplished without further soluble factors such as anti-inflammatory peptides^48^ or additional cell types such as mesenchymal stromal cells.^74−76^ These differences could be attributed to differences in cytokine binding to negatively charged sulfated GAGs (sGAGs) compared to collagen alone, as demonstrated by the increased binding of IL-1β to sGAG gels. By binding to sGAG chains, IL-1β is effectively hidden from the encapsulated cells, preventing the cytokine from interacting with the cell surface and activating the pro-inflammatory cascade. This would be consistent with similar negatively charged materials that have also demonstrated anti-inflammatory effects on encapsulated cells.^53^ However, further testing is required, particularly with regard to cellular mechanisms of inflammation (e.g., toll-like receptor activation and nuclear factor-κβ transnuclear localization). For these GAG hydrogels, the presence of collagen type I also did not appear to have a large effect on the encapsulated cells but did provide the gels with resistance to swelling and retention of compressive strength over time. Although the gels did not completely prevent a response to inflammatory stimuli, as seen by the increased IL-6 production and decreased expression of certain cartilage matrix protein genes, the GAG gels still performed better than the collagen-only controls, with the production of IL-8 and CCL2 being comparable to the unstimulated groups for most GAG groups. These findings are consistent with those of previous studies involving sulfated hydrogels in the suppression of inflammation. Arlov et al. found that the sulfation of alginate suppressed, but did not completely prevent, IL-1β-mediated inflammation over the course of 2 days when using lower doses of IL-1β than that used here.^53^ While additional work is required to better determine the mechanism of action of inflammation suppression, whether through interactions between the GAGs and the encapsulated cells or through the sequestration of IL-1β, this study provides an early indication that GAG gels present a good foundation to design constructs that support cartilage regeneration in an inflammatory environment like that seen in joints afflicted with OA. Further studies are needed to determine the optimal way to promote the generation of new cartilage using these methods to attenuate induced inflammation.

With the development of these chemically cross-linked sGAG-based hydrogels and the results of this study, subsequently developed materials could potentially be used as in situ gelling hydrogels for the filling of focal-sized chondral defects, where autologous cells could be isolated and reimplanted into the defect in cases of osteoarthritis. In these cases, low-grade inflammation of the joint is already apparent, limiting the growth of new cartilage.^77^ Once implanted, the material protects the cells from the pro-inflammatory environment, allowing for the growth of new tissue without being inhibited by inflammation. By regenerating the cartilage, intensive procedures including total joint arthroscopy can be avoided. Further studies will build upon this work by determining the optimal way to promote the generation of new cartilage following implantation and using these methods to attenuate induced inflammation to maximize cartilage regeneration.

## 5 Conclusions

In this study, we demonstrated the ability of blended CS/HA hydrogels to dampen the inflammatory response of encapsulated chondrocytes when cultured with a sustained dose of IL-1β for 14 days. Under these pro-inflammatory conditions, the GAG gels maintained similar robustness to their unstimulated counterparts, and the encapsulated cells showed signs of reduced inflammation-induced changes in cell proliferation. Furthermore, although cells still produced some levels of IL-6 and showed some upregulation of MMP13 and downregulation of cartilage matrix proteins, chondrocytes cultured in the GAG gels were less susceptible to inflammation than cells in the collagen-only gel control. Based on the results of this study, the observed anti-inflammatory effects may be due to the bioactive signals from the GAGs themselves, sequestration of IL-1β from the cells through GAG/protein interactions, or a combination of the two. Altogether, GAG gels possess the potential to allow cartilage tissue engineering and engineering of additional tissue types under pro-inflammatory conditions.

## Supporting Information Available

Standard curve for quantification of thiols; thiol quantification of CS and HA; binding of IL-1β to hydrogels; MMP13 activity of supernatant from gels cultured with IL-1β; DNA content in gels after 14 days; normalized metabolic response after 14 days; immunofluorescence images of aggrecan, collagen, and nuclei in 3:7 CS/HA GAG and collagen gels with and without IL-1β; and quantification of immunofluorescence images (PDF)
