# Supplementary material for: Chondroitin Sulfate/Hyaluronic Acid-Blended Hydrogels Suppress Chondrocyte Inflammation under Pro-Inflammatory Conditions
Source: ACS Biomater Sci Eng. 2024 Apr 18;10(5):3242–54. doi: 10.1021/acsbiomaterials.4c00200 (PMC11094685; doi:10.1021/acsbiomaterials.4c00200)
Supplement: Supplementary file 1 — ab4c00200_si_001.pdf [file ab4c00200_si_001.pdf]

Supporting Information

Chondroitin Sulfate/Hyaluronic Acid Blended Hydrogels Suppress Chondrocyte Inflammation under Pro-Inflammatory Conditions

Author Names: Michael Nguyen[a], Carly M. Battistoni[b], Paulina M. Babiak[b], Julie C. Liu[b,c,#], Alyssa Panitch[a,d*,#]

Author Address:

[a] Department of Biomedical Engineering, University of California, Davis, USA

[b] Davidson School of Chemical Engineering, Purdue University, West Lafayette, IN, USA

[c] Weldon School of Biomedical Engineering, Purdue University, West Lafayette, IN, 47907, USA

[d]Wallace H. Coulter Department of Biomedical Engineering, Georgia Institute of Technology and Emory University, GA, USA

*Corresponding Author, alyssa.panitch@bme.gatech.edu

[#]Co-senior authors

Thiolated CS and Thiolated HA Characterization and Quantification

Free Thiol Quantification by Ellman's Assay

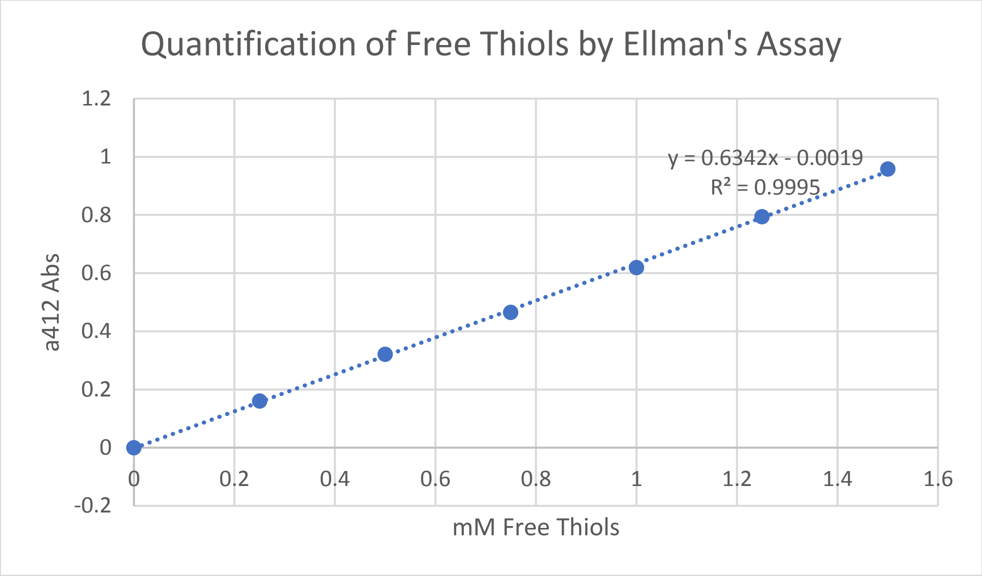

Figure S1: Standard Curve for Quantification of Free Thiols Using Ellman's Reagent

Table S1: Free Thiol Quantification of CS-SH and HA-SH

	Average 412 nm	Average mM -SH	Average Degree of Thiolation
CS-SH – 40 kDa MW	0.239	0.380	18.12 %
HA-SH – 100 kDa MW	0.306	0.485	18.4 %

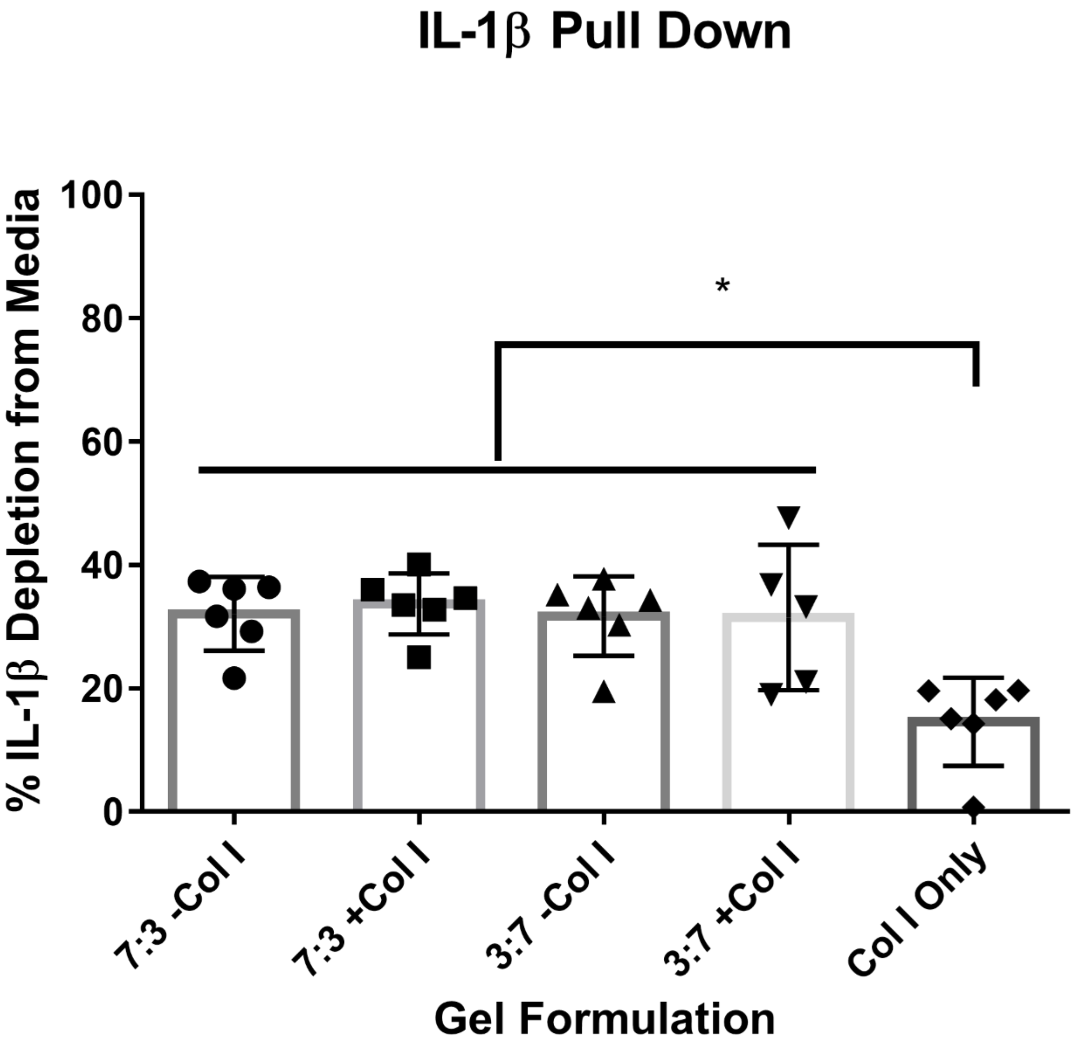

Figure S2: Binding of media Il-1β to GAG and collagen only hydrogels

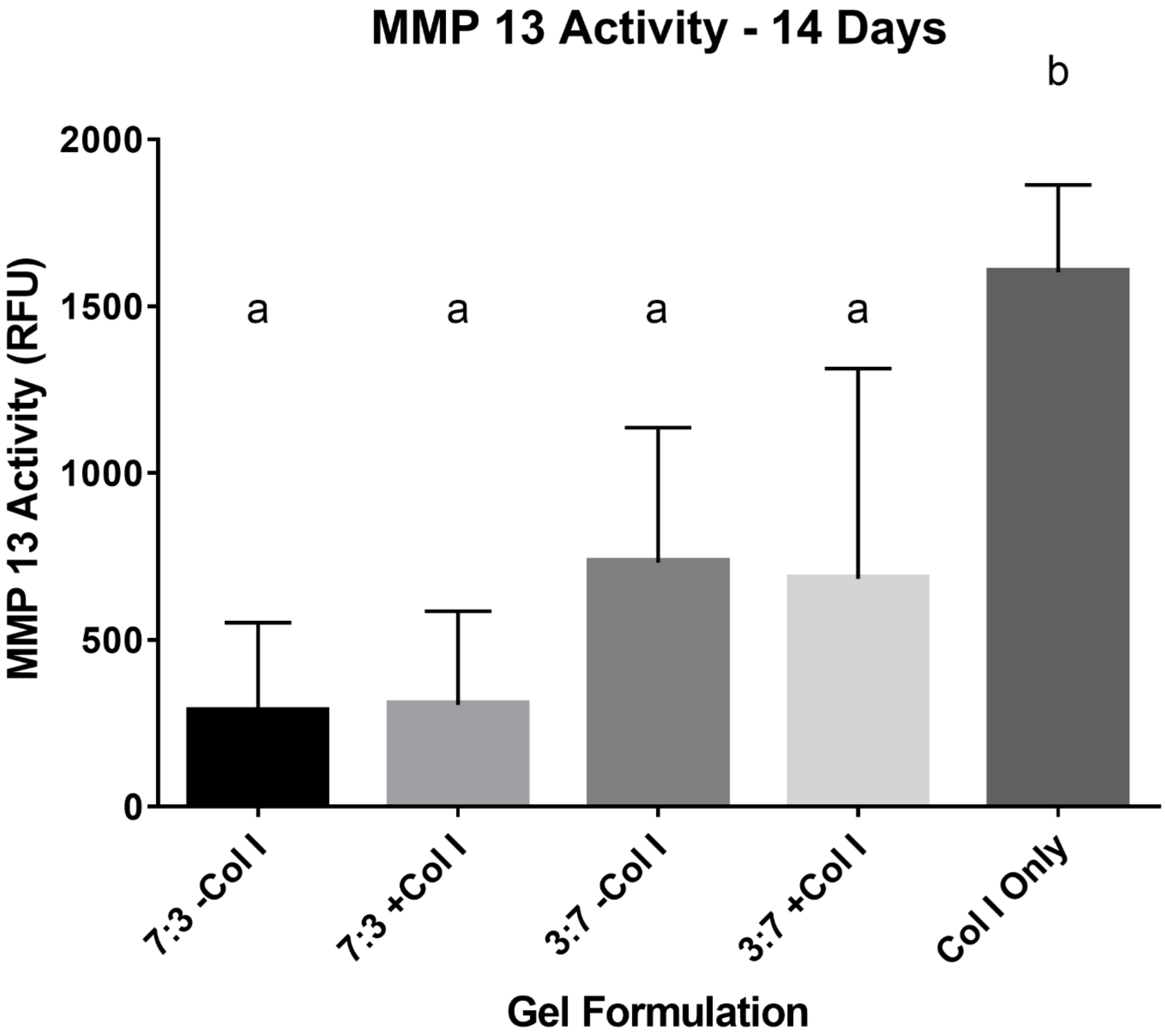

Figure S3: Activity of MMP13 in media of groups stimulated with IL-1β. Groups that share letters are not statistically significant from each other ($P > 0.05$). Groups that do not share letters are statistically significant from each other ($P < 0.05$)

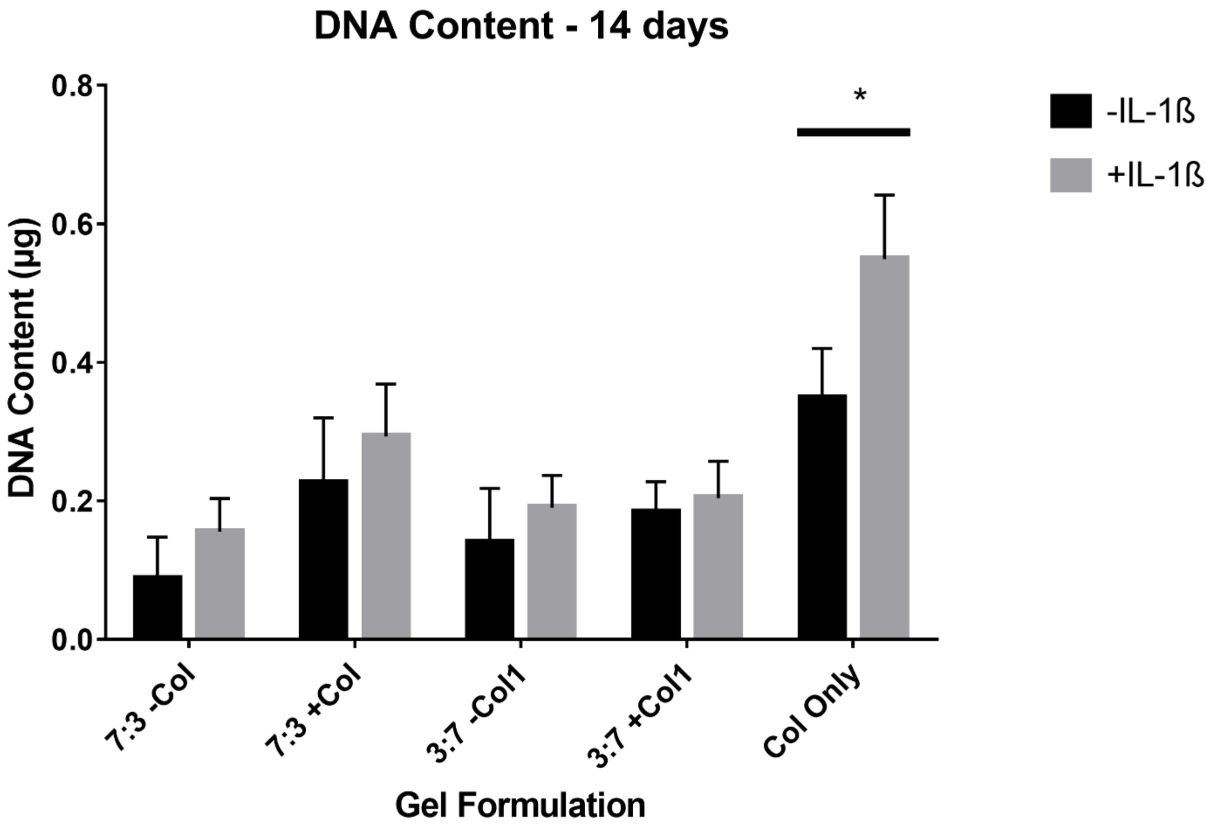

Figure S4: Changes in cellular DNA content contained in cultured hydrogels after fourteen days

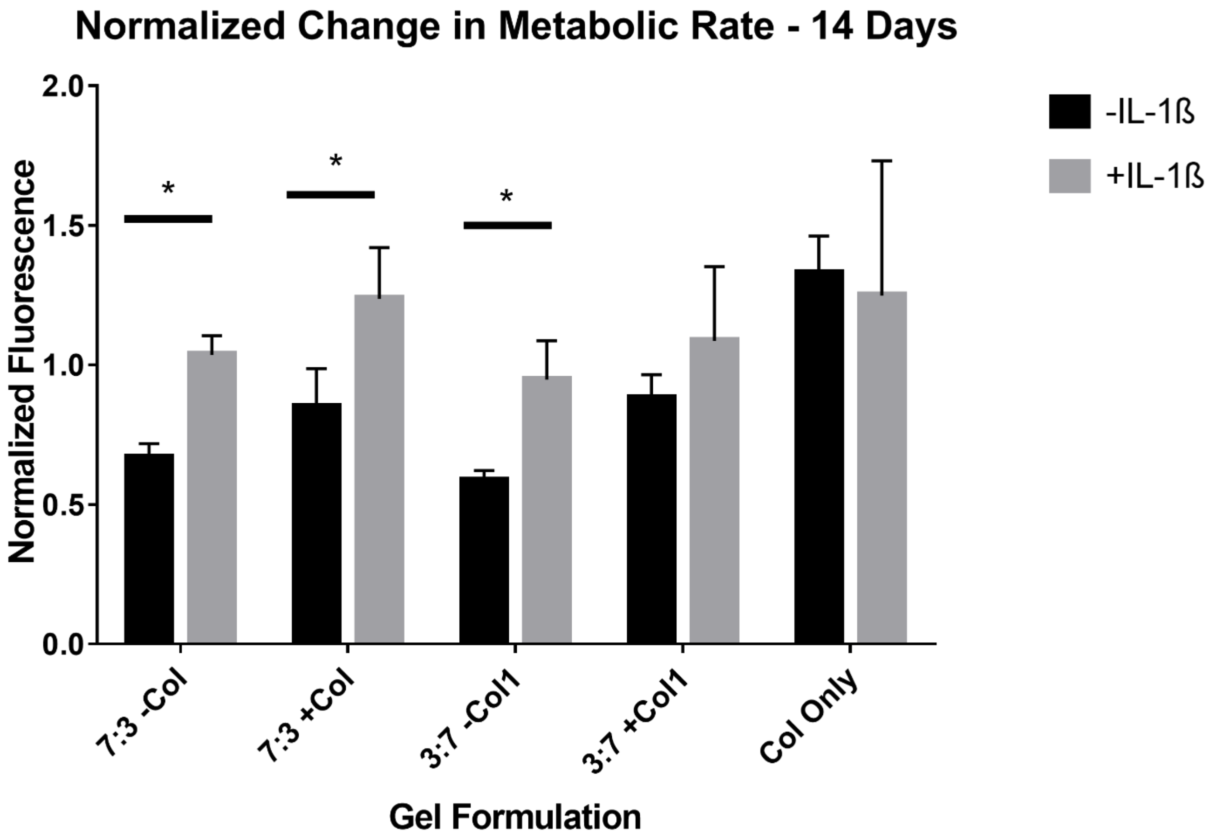

Figure S5: Changes in cell metabolic rate in response to stimulation with IL-1β. * Indicates groups are statistically significant from one another (P < 0.05)

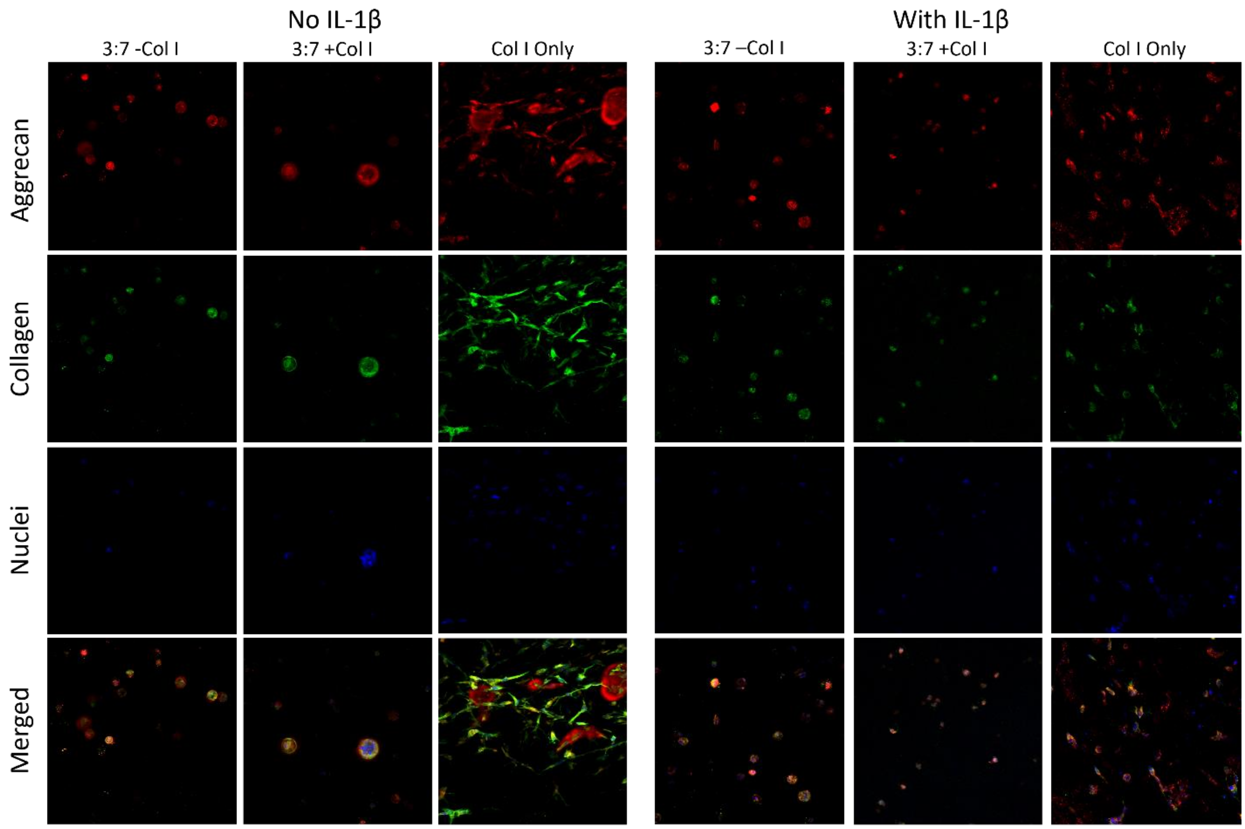

Figure S6: Immunohistochemical staining of aggrecan and collagen II produced by fbACs cultured in 3:7 CS/HA GAG and collagen gels with and without Il-1β. Images are composed of a maximum projection Z-stack of a 300 µm thick sample section, with images taken every 20 µm. Scale bar represents 50 µm. Images for Col I only gels is reproduced from figure 5 in the main text

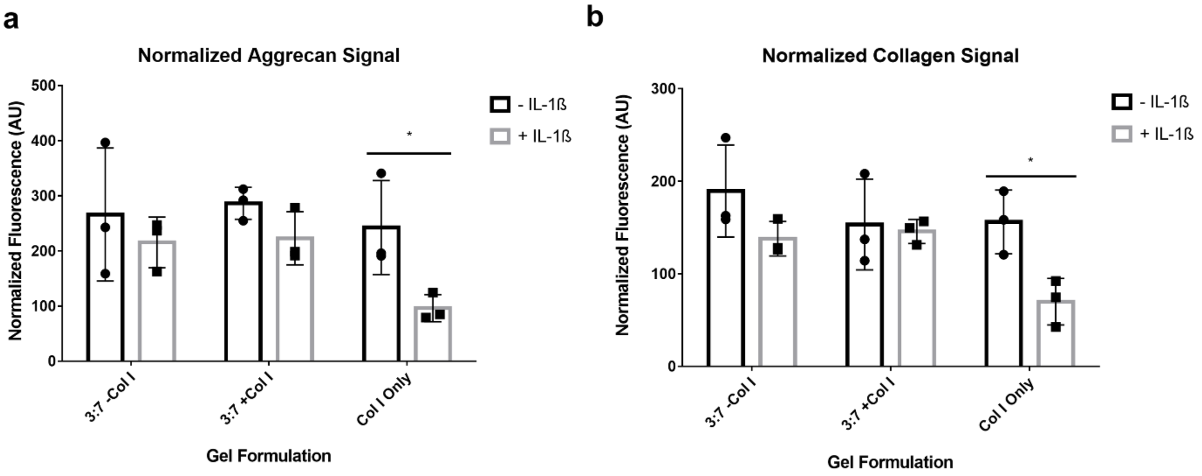

Figure S7: Quantification of immunohistochemical staining of aggrecan and collagen produced by fbACs cultured in GAG and collagen gels with and without IL-1β. * denotes statistical significance ($P < 0.05$) between gels cultured with and without IL-1β. Data for Col I only gels is reproduced from figure 6 in the main text.
